# *Aspergillus* Pericarditis with Tamponade in a Renal Transplant Patient

**DOI:** 10.1155/2017/7134586

**Published:** 2017-02-20

**Authors:** Sylvia Biso, Rapeepat Lekkham, Antoinette Climaco

**Affiliations:** Albert Einstein Medical Center, 5501 Old York Road, Philadelphia, PA 19141, USA

## Abstract

*Aspergillus* pericarditis is a rare and life-threatening infection in immunosuppressed patients. It has nonspecific clinical manifestations that often mimic other disease entities especially in patients who have extensive comorbidities. Diagnosis is oftentimes delayed and rarely done antemortem. A high degree of suspicion in immunocompromised patients is necessary for evaluation and timely diagnosis. This is a case of* Aspergillus* pericarditis with cardiac tamponade in a renal transplant patient with liver cirrhosis. Two months after transplant, he developed decompensation of his cirrhosis from hepatitis C, acute cellular rejection, and* Kluyvera* bacteremia, followed by vancomycin-resistant* Enterococcus faecium* (VRE) bacteremia. Four months after transplant, the patient presented with lethargy and fluid overload. He subsequently developed shock and ventilator-dependent respiratory failure. An echocardiogram showed pericardial effusion with cardiac tamponade. He had emergent pericardiocentesis that showed purulent drainage. He was started on broad-spectrum antibiotics. Amphotericin B was initiated when the pericardial fluid grew mold that was later identified as* Aspergillus fumigatus*. The patient quickly decompensated and expired.

## 1. Introduction

Pericardial aspergillosis is a fatal opportunistic infection in immunosuppressed patients. It is oftentimes difficult to diagnose because of the lack of characteristic signs and symptoms. Risk factors for predisposing for this infection include transplant patients, neutropenic, AIDS, and chronic granulomatous disease patients, and patients receiving steroids or immunosuppressive regimens [1]. The most common site of infection is the lung and it accounts for 70% of the cases. This reflects the lungs as the port of entry. In a study by Lin et al., the distribution of patients according to site or type of aspergillosis is as follows: disseminated or CNS infections (9%), sinusitis (2.7%), tracheobronchitis (2.3%), multisite (5.9%), and cutaneous, other, or unspecified (10.3%) [2]. Cardiac aspergillosis is uncommon and often occurs as endocarditis or myocarditis. Isolated pericardial involvement is rare and not usually diagnosed antemortem. In a study of 60 patients with fungal cardiac infections, the most common cause was* Candida* (62%).* Aspergillus* and phycomycetes were second at 12% each [3]. We report a case of* Aspergillus* pericarditis with tamponade in a renal transplant patient with liver cirrhosis.

## 2. Case Report

The patient is a 61-year-old male with end stage renal disease from hypertension with cadaveric renal transplant recipient who developed acute decompensated liver cirrhosis from viral hepatitis C after transplant.

Two months after transplant, he had several episodes of gastrointestinal bleeding, urinary tract infections, and cardiac arrest with acute allograft dysfunction requiring renal replacement therapies. His mycophenolate mofetil was held. The transplant kidney biopsy showed evidence of acute cellular rejection with acute tubular necrosis that was treated with high-dose steroids. However, his allograft function never recovered and he required supportive hemodialysis. He also developed* Kluyvera* bacteremia followed by VRE bacteremia.

Four months after transplant, the patient presented with altered mental status and severe volume overload. On admission, the patient was lethargic, afebrile, and normotensive but tachycardic. Physical examination revealed severe anasarca and asterixis with no focal deficit. Diagnosis of metabolic encephalopathy was entertained. Urgent hemodialysis for uremia was started. His lactulose was increased for presumed hepatic encephalopathy without any improvement.

On the 5th hospital day, the patient suddenly became unresponsive, hypotensive with shock, hypoxic, and febrile. He was intubated and started on vasopressors. Chest X-ray showed left lower lobe consolidation, but did not reveal cardiomegaly. His ECG showed diffuse ST segment elevations. PR segment depressions, electrical alternans, and low QRS voltage were absent. Transthoracic echocardiogram showed moderate size pericardial effusion localized circumferentially. This was accompanied by right ventricular compression with tamponade physiology. The patient had emergent pericardiocentesis with pericardial drain placement. A total of 320 ml of purulent fluid was drained. His blood pressure improved and he was taken off vasopressors. He was also started on daptomycin and cefepime.

**Figure 1:**
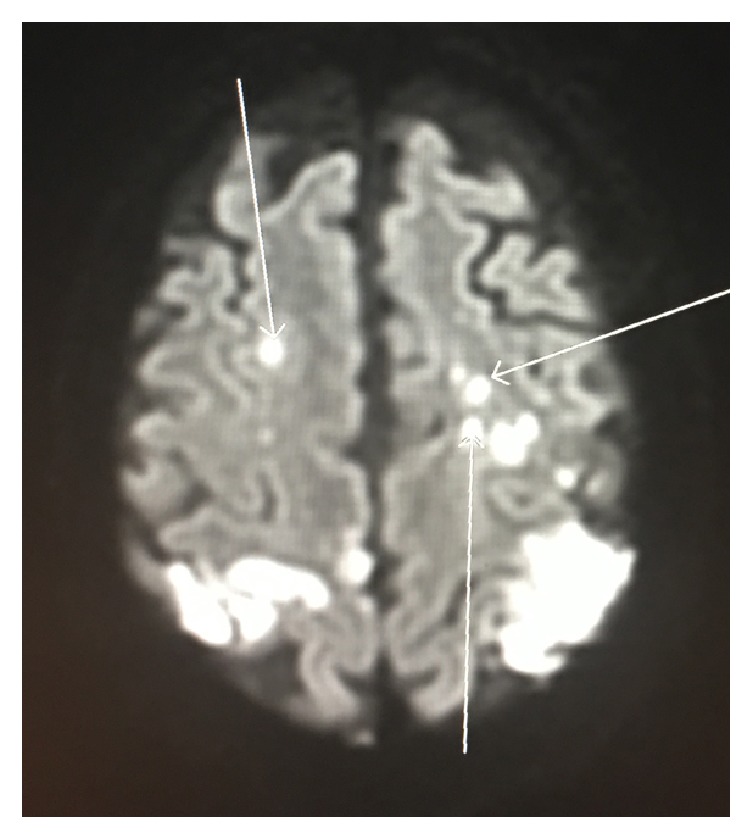
Brain MRI showing multiple septic emboli.

**Figure 2:**
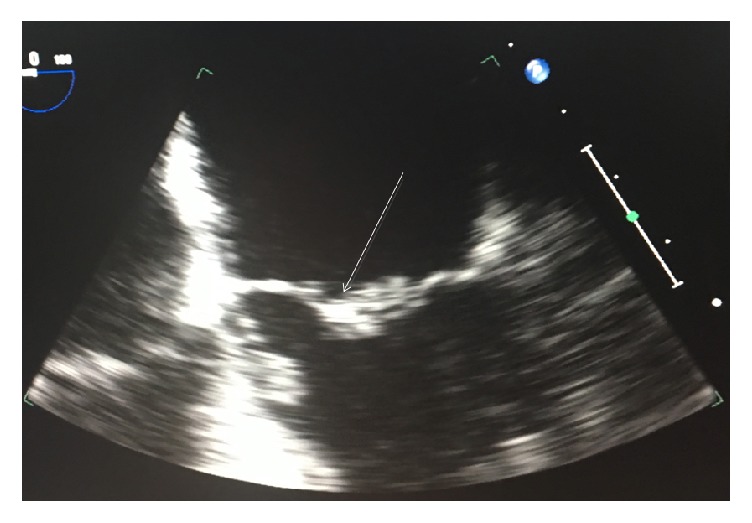
TEE showing possible small mitral valve vegetations.

On the 6th hospital day, the patient continued to be lethargic. A CT scan of the head showed evidence of multifocal infarctions. Subsequent MRI of the brain revealed multiple septic emboli (Figure 1). These results raised suspicion of infective endocarditis for which a transesophageal echocardiogram (TEE) was performed. TEE showed possible small mitral valve vegetations (Figure 2) and resolution of the pericardial effusion. Blood cultures grew vancomycin-resistant* Enterococcus faecium*. The hemodialysis catheter was changed over the wire and culture of the catheter tip was subsequently found to be negative. Cefepime was switched to ceftaroline for better daptomycin binding to bacterial membrane.

On the 7th hospital day, mold grew in pericardial fluid culture. Amphotericin B was initiated to cover both* Aspergillus* and* Mucor*. The patient continued to clinically deteriorate and went into septic shock. The family gave him a do-not-resuscitate status and he expired on the 7th hospital day. The mold was eventually identified as* Aspergillus fumigatus* 10 days after the patient died.

## 3. Aspergillosis

Cardiac aspergillosis remains a diagnostic challenge especially without prior established pulmonary aspergillosis. A high degree clinical suspicion on an immunocompromised patient is needed to direct the clinical investigation. There are currently thirty-two cases of pericardial aspergillosis found in literature. Eight had clinical signs of cardiac tamponade. Only three, including our case, did not have established pulmonary or other forms of aspergillosis. Most diagnoses were postmortem. Only six out of the thirty-two were cured.

Among patients predisposed to aspergillosis, cancer is the most common underlying medical condition (44%), followed by bone marrow transplant (25%), solid-organ transplant (13%), HIV/AIDS (3.8), autoimmune (2%), and systemic steroid use (3.5%). Our patient is a renal transplant patient with concurrent immunosuppressant use. His clinical course was complicated by acute decompensated liver cirrhosis, acute renal allograft dysfunction, and severe infections within two months of renal transplant. These extensive comorbidities made him susceptible to opportunistic infections. Four months after his renal transplant, he had aspergillosis pericarditis with tamponade that eventually led to his demise.

Weiland et al. [4] studied the clinical pattern of aspergillosis in renal transplant patients. The median time between aspergillosis infection and transplantation was found to be three and a half months. For patients with history of acute rejection, aspergillosis occurred within two months of treatment of acute rejection. These data are consistent with the clinical course of our patient. Weiland et al. also found that aspergillosis took place alongside other illnesses. The most common associated infection was CMV followed by bacterial infections. One-fourth of patients affected were also multiple transplant recipients. Weiland et al. concluded that for renal transplant patients, patients with CMV, recent acute rejection and recipients of multiple transplants appear to be more susceptible to aspergillosis.

The clinical features of cardiac aspergillosis are nonspecific and often depend on which part of the heart is involved. Myocardial aspergillosis is the most common form of the infection and can account to up to 83% of cases. It may occur alone but often with endocarditis or pericarditis. It is often asymptomatic, although it can manifest as a conduction abnormality and is usually diagnosed during autopsy [5].

Aspergillosis endocarditis occurs in 17% of patients and presents as fever, embolic episodes, or heart murmur. Unlike* Aspergillus* endocarditis where large vegetations are easily seen in echocardiography, myocardial or pericardial aspergillosis are harder to detect [5]. Our patient had septic emboli to the brain, a frequent sequelae of endocarditis. Transesophageal echocardiogram did reveal possible small valvular vegetations. This shows that the patient may have accompanying infective endocarditis. Embolic events in the setting of aspergillosis without significant valvular vegetations may also signify mural endocarditis [6].* Aspergillus* mural endocarditis is not always apparent in echocardiograms and may start off as a subendocardial focus before developing into an abscess [7].

*Aspergillus* is difficult to grow in blood cultures. Our hospital uses Bactec Fx culture system and requires 5–7 ml of blood sample per culture bottle. In this patient, a bottle of blood culture on admission did not grow any organism. On day 5, when the patient was clinically deteriorating, repeat blood cultures consisting of two bottles only grew* Enterococcus faecium*. On day 6, repeat blood cultures again showed no growth in one bottle and* E. faecium* in the second bottle. It was only in the pericardial fluid fungal culture collected on day 5 that* Aspergillus fumigatus* was demonstrated and grown.

*Aspergillus* pericarditis occurs in multisite infections and is seen in 17% of cardiac aspergillosis [5]. It presents as chest pain, hypotension, tamponade, or pericardial friction rub. In our patient, diffuse ST segment elevations and hypotension pointed towards cardiac tamponade. Before this, his lethargy was attributed to metabolic encephalopathy. The patient, however, quickly deteriorated and eventually expired.

The initial therapy for invasive aspergillosis is voriconazole, a broad-spectrum triazole antifungal. It has been shown to be more effective than amphotericin B and is associated with lower mortality rate [8]. Our patient, on the other hand, was started on amphotericin B when pericardial fluid revealed mold. This is to cover for both* Aspergillus* and* Mucor*.* Mucor* is another mold infection that occurs in transplant patients and voriconazole does not have anti-*Mucor* activity. Furthermore, for patients who have liver disease like ours or for patients who cannot tolerate voriconazole, a lipid formulation of amphotericin B is the next alternative to voriconazole.

Other treatment strategies involve combination therapy of voriconazole and echinocandins. The combination therapy may be used as primary treatment or as salvage therapy. The dual therapy was shown to have superior outcomes than voriconazole alone in one study [9]. Echinocandins, however, are not used as initial monotherapy of aspergillosis. They can be used as salvage therapy for those who cannot tolerate or are refractory to triazoles.

Invasive aspergillosis portends a poor prognosis and has a case fatality rate (CFR) of 58%. The CFR for liver transplant patients is 67.6, while kidney transplant patients' CFR is 62.5% [2]. For pericardial aspergillosis, only six out of thirty-two patients of the cases published in literature have been cured. Factors that contributed to the survival of the six patients were early detection, recovery from immunosuppression, pericardiectomy, and combination antifungal therapy [10].

In conclusion, pericardial aspergillosis is a rare and fatal disease in immunosuppressed patients. It is often insidious and has nonspecific clinical manifestations. A high degree of clinical suspicion is required for early detection and diagnosis. Once diagnosed, aggressive treatment is warranted.
